# Comparison of alterations in cerebral hemoglobin oxygenation in late life depression and Alzheimer’s disease as assessed by near-infrared spectroscopy

**DOI:** 10.1186/1744-9081-10-8

**Published:** 2014-03-17

**Authors:** Hisashi Kito, Akiko Ryokawa, Yoshihiro Kinoshita, Daimei Sasayama, Nobuhiro Sugiyama, Tomomi Ogihara, Takehiko Yasaki, Tetsuya Hagiwara, Shin Inuzuka, Tohru Takahashi, Hirokazu Genno, Hiroshi Nose, Tokiji Hanihara, Shinsuke Washizuka, Naoji Amano

**Affiliations:** 1 Department of Psychiatry, Shinshu University School of Medicine, 3-1-1 Asahi, Matsumoto, Nagano 390-8621, Japan; 2 Department of Sports Medical Sciences, Shinshu University Graduate School of Medicine, 3-1-1 Asahi, Matsumoto, Nagano 390-8621, Japan; 3 School of Health Sciences, Shinshu University School of Medicine, 3-1-1 Asahi, Matsumoto, Nagano 390-8621, Japan

**Keywords:** Near-infrared spectroscopy, Brain activation, Alzheimer’s disease, Depression

## Abstract

**Background:**

Patients with Alzheimer’s disease (AD) often present with apathy symptoms resembling the decreased motivation observed in depressed patients. Therefore, differentiating the initial phase of AD from late life depression may be difficult in some cases. Near-infrared spectroscopy (NIRS) is a functional neuroimaging modality that uses near-infrared light to measure changes in hemoglobin concentration on the cortical surface during activation tasks. The objective of this study was to investigate differences in brain activation associated with late life depression and with AD by means of NIRS.

**Methods:**

NIRS was performed in 30 patients with depression, 28 patients with AD, and 33 healthy controls, all aged 60 years or older. During two tasks, a verbal fluency task and a visuospatial task, changes in oxygenated hemoglobin concentration in the frontal and parietal cortices were investigated.

**Results:**

In the visuospatial task, cortical activation was lower in the depressed group than in the AD group, and significant differences were observed in the parietal cortex.

**Conclusions:**

NIRS can detect differences in brain activation between patients with late life depression and those with AD. NIRS is a promising tool for the differential diagnosis of late life depression and AD.

## Background

Apathy in dementia is often difficult to distinguish from decreased motivation in depressed patients. Therefore, careful observation of the clinical course may be required to differentiate late life depression from the initial phase of Alzheimer’s disease (AD). Depression-related and age-related factors mutually affect each other. For this reason, late life depression tends to be accompanied by cognitive dysfunction and clinical symptoms are multifaceted, making diagnosis difficult. To discriminate depression from dementia, brain imaging may be a helpful addition to a detailed examination of the patient’s medical history and current findings. For this purpose, near-infrared spectroscopy (NIRS) may offer a means to aid the differential diagnosis of these conditions.

NIRS, a method for measuring brain function that was developed during the 1990s, is used to measure changes in regional cerebral blood flow (rCBF) associated with brain activation. Using near-infrared light, NIRS can detect rCBF changes as represented by increases in oxygenated hemoglobin concentration ([oxy-Hb]) and decreases in deoxygenated hemoglobin concentration ([deoxy-Hb]), which are considered to reflect cortical activity [1]. It has been reported that the results of NIRS correlate strongly with those of functional magnetic resonance imaging (fMRI) [2]. The advantage of NIRS over other functional neuroimaging techniques such as fMRI is that subjects can be examined more easily in the sitting position.

To date, several NIRS studies have compared findings in patients with AD with those in healthy controls. In AD, Herrmann *et al.*[3] reported a small increase in [oxy-Hb] in the prefrontal cortex during a verbal fluency task (VFT), while Arai *et al.*[4] reported decreased brain activation in both the frontal and parietal lobes during a VFT. In addition, Zeller *et al.*[5] found activation deficits in the parietal cortex during a visuospatial task. On the other hand, in NIRS studies of late life depression, Matsuo *et al.*[6] reported significantly less activation of the prefrontal cortex in AD, and several other studies have reported hypofrontality [7-9]. However, we are not aware of any studies that have used NIRS for a direct comparison of late life depression and AD. We believe that such a comparison is of clinical significance, and could potentially aid in the differential diagnosis of late life depression and AD.

Neuroimaging studies comparing the two conditions include a report on single photon emission tomography which revealed markedly decreased rCBF in the temporal and parietal lobes in AD, while in the frontal lobe, rCBF was markedly decreased in depression [10]. Previous fMRI studies have shown inconsistent results regarding the hemodynamic response in AD or depression, and none have directly compared the activation patterns of both conditions [11-18].

The objective of the present study was to investigate differences in brain activation between late life depression and AD by means of NIRS. We examined activation in the frontal and parietal cortices during two activation tasks, a VFT and a visuospatial task, in patients with AD or depression in comparison with healthy subjects as controls. Given that several lines of evidence suggest the involvement of executive deficits associated with frontal lobe dysfunction in the pathophysiology of depression [19,20] and that both frontal and parietal dysfunctions are observed from the initial phase of AD [21-24], we hypothesized that activation in the parietal cortex would show a more marked decrease in patients with AD than in those with depression.

## Methods

### Participants

Participants were male and female patients aged 60 years or older receiving inpatient or outpatient care at the Department of Psychiatry, Shinshu University Hospital, Japan. In total, 33 patients met the Diagnostic and Statistical Manual of Mental Disorders-IV diagnostic criteria for depression and 32 other patients met that for AD. Patients who met the diagnostic criteria for both depression and AD were excluded from the study. All patients had been diagnosed by psychiatrists. In addition, 33 healthy subjects aged 60 years or over who were members of municipal physical fitness clubs, who had no history of psychiatric or neurological disease, and were no taking psychotropics were enrolled as controls (Table 1). All participants were right-handed. All patients were taking psychotropic medication. Patients with depression were treated with antidepressants (5 with amitriptyline, 1 with maprotiline, 6 with sertraline, 2 with fluvoxamine, 3 with escitalopram, 10 with duloxetine, 2 with milnacipran, 3 with mirtazapine, and 1 with mianserin), and patients with AD were treated with anti-dementia drugs (27 with donepezil, 4 with memantine, and 1 with galantamine). Head MRI was conducted for all patients, and those with findings that would affect rCBF, such as gross cerebral infarction or cerebral aneurysm, were excluded. Also excluded were patients with endocrine, metabolic, or neurological disorders.

**Table 1:** Clinical data

	**Controls**	**Depression**	**Alzheimer’s Disease**	**Statistics**	***Post hoc *** **comparisons**		
	**(n = 33)**	**(n = 30)**	**(n = 28)**		**D vs AD**	**D vs HC**	**AD vs HC**
Male:Female	11:22	9:21	10:18	χ2 = 0.22, df = 2, p = 0.90			
Age (years)	69.6 ± 5.5	71.1 ± 6.8	76.6 ± 6.9	F = 9.66 p < 0.001	p = 0.005	p = 0.603	p < 0.001
MMSE score	29.2 ± 1.2	26.6 ± 2.3	21.6 ± 4.4	F = 52.7 p < 0.001	p < 0.001	p = 0.002	p < 0.001
FAB score	16.1 ± 1.2	14.1 ± 2.7	12.0 ± 3.2	F = 20.1 p < 0.001	p = 0.008	p = 0.005	p < 0.001
CDR score	0	0.05 ± 0.2	0.8 ± 0.4	p < 0.001	p < 0.001	p = 0.065	p < 0.001
HAMD score	0.2 ± 0.8	10.7 ± 8.9	4.4 ± 7.1	F = 20.3 p < 0.001	p = 0.001	p < 0.001	p = 0.037
VFT performance	41.3 ± 7.9	33.2 ± 12.2	33.9 ± 10.1	F = 6.19 p = 0.003	p = 0.963	p = 0.006	p = 0.015
Benton Judgment of Line Orientation performance	22.6 ± 6.6	12.5 ± 4.2	13.7 ± 8.5	F = 21.6 p < 0.001	p = 0.779	p < 0.001	p < 0.001

Continuous values are shown as the means ± standard deviation. *Abbreviations*: *AD* Alzheimer’s disease, *CDR* Clinical Dementia Rating, *D* depression, *df* degrees of freedom, *FAB* Frontal Assessment Battery, *HAMD* Hamilton Rating Scale for Depression, *HC* healthy controls, *MMSE* Mini Mental State Examination, *χ*^*2*^ Chi-squared value.

### Ethics

This study was approved by the Ethics Committee of Shinshu University School of Medicine (No. 1488), and all participants provided written informed consent. For patients with AD, written consent was also obtained from their guardians.

### Clinical evaluation

All participants were assessed using the 21-item Hamilton Rating Scale for Depression (HAMD) [25], Clinical Dementia Rating (CDR) [26], Mini Mental State Examination (MMSE) [27], and Frontal Assessment Battery (FAB). The FAB assesses decreases in frontal lobe function and comprises 6 test items [28]. All tests were conducted by a single trained clinical psychotherapist.

### NIRS measurements

Changes in [oxy-Hb] and [deoxy-Hb] were measured using an NIRS system (FOIRE-3000; Shimadzu Corporation, Kyoto, Japan). Absorption was measured at three wavelengths of near-infrared light (780, 805, and 830 nm), and [oxy-Hb] and [deoxy-Hb] levels were calculated according to the Lambert-Beer law based on the differences in absorption between the three wavelengths. The NIRS system can measure changes in hemoglobin concentrations at a range of approximately 2-3 cm from the surface of the skull. The distance between each pair of emission and detector probes was 3.0 cm, and the measurement area between each pair was defined as a ‘channel (CH)’. In total, 22 channels were positioned in a 6 × 12 cm area covering the frontal and parietal cortices, with CH 1-22 corresponding to the frontal cortex and CH 23-44 to the parietal cortex. The lowest probes in the frontal cortex were positioned along the Fp1-Fp2 line according to the International 10-20 system. Probes for the parietal cortex were positioned such that Pz was located at the center.

### Activation tasks

The NIRS study was performed with the participants sitting relaxed in a chair and facing the screen of a desktop computer. NIRS measurement was performed using a block design. A 30-s rest period was established, followed by a 60-s task period and another 30-s rest period. This sequence was repeated 3 times in succession. Based on previous studies [5,29], we applied the VFT and a visuospatial task (Benton Judgment of Line Orientation). The latter is a standardized test assessing the ability to recognize the angle of two lines, and it has been reported that task performance is decreased in AD [30]. A 2-3-min interval was established between the VFT and visuospatial task. During the rest period of the VFT, participants repeated the Japanese syllables “a, i, u, e, o” (corresponding to “A, B, C…” in English) as a control. During the task period, participants were asked to respond with as many words as possible beginning with the Japanese morae (syllables) “ki”, “o”, “i”, “sa”, “ta”, “a”, “ku”, “u”, and “ko”. The task was performed starting with words beginning with “ki”, and oral instructions were given every 20 s to change to words beginning with the next mora. Participants were asked to perform an active control task during the rest period so that the NIRS signal caused by vocalization could be subtracted from the NIRS signal during the task period. Task performance was scored as the number of words spoken during the VFT. In the visuospatial task, participants were asked to repeat 11 random numbers displayed on the screen during the rest period. Then, during the task period, the Benton Judgment of Line Orientation was displayed on the computer, with the next task displayed after the subject responded. Task performance was scored as the number of correct responses. While these tasks were being performed, changes in hemoglobin concentrations were measured using the 44-channel NIRS.

### Data analysis

Clinical data were compared between controls, depressed patients, and patients with AD by analysis of variance (ANOVA) or the Krusksal-Wallis test for continuous variables and the Chi-squared test for categorical variables. *Post hoc* comparisons were performed using Tukey’s honestly significant difference test for ANOVA and the Mann-Whitney U-test for the Kruskal-Wallis test.

NIRS data with significant artifacts were excluded from the analysis. Thus, data from 30 patients with depression, 28 patients with AD, and 33 healthy controls were analyzed. Although [oxy-Hb], [deoxy-Hb], and total hemoglobin concentrations can be measured by NIRS, we concentrated on [oxy-Hb], which correlates best with changes in rCBF [31]. After averaging the results of three consecutive runs of the same task for each participant, a baseline correction was performed setting the amount of change in [oxy-Hb] at the start of the task as zero. We defined cortical activation as the difference between average [oxy-Hb] during the pre-task period and that during the task period. NIRS measurements were the product of the change in hemoglobin concentration and the optical path length; however, the optical path length differed according to measurement site. Consequently, because all channels were independent, the cortical activation in each of the 44 channels was comparatively analyzed. Since none of the cortical activation data followed a normal distribution as determined by the Shapiro-Wilk test, the data were compared between the three groups using the Kruskal-Wallis test. *Post hoc* comparisons between the groups were performed using the Mann-Whitney U-test. Because data were analyzed for 44 channels, the false discovery rate (FDR) was used to correct for multiple comparisons [32]. A corrected p-value of less than 0.05 was considered significant. Spearman’s rank correlation coefficient was used to analyze correlations between clinical data and the cortical activation observed in each channel, and FDR was used to correct for multiple comparisons. Stepwise logistic regression analysis was performed for patients with depression and those with AD to determine the optimal model for predicting patients with depression. Stepwise analysis was conducted as a forward stepping procedure based on a likelihood ratio test, with p < 0.05 for variable inclusion and p > 0.1 for variable exclusion from the model. The cortical activations of the 44 channels were used as potential predictor variables. Nagelkerke’s coefficient of determination was used to approximate the percent of variance explained by the model [33]. The area under the receiver operating characteristic curve (AUC) was also used to determine the predictive power of the logistic model. The predicted probability with the highest Youden index was selected as the optimal cut-off point. SPSS software (Version 20.0) was used for statistical analysis.

## Results

### Clinical data

MMSE and FAB scores were decreased in the order of healthy controls, patients with depression, and patients with AD, confirming a decrease in cognitive function (Table 1). MMSE and CDR scores confirmed that the patients with AD were in the initial phase of the disease. HAMD scores indicated the severity of depression was mild [34]. Task performance in both patient groups was not significantly different (p = 0.963 in the VFT and p = 0.779 in the Benton Judgment of Line Orientation). The results of head MRI showed temporal and parietal lobe atrophy in all patients with AD. No patients with depression presented notable pathological findings.

### NIRS data

#### General cortical activation patterns

**Figure 1:**
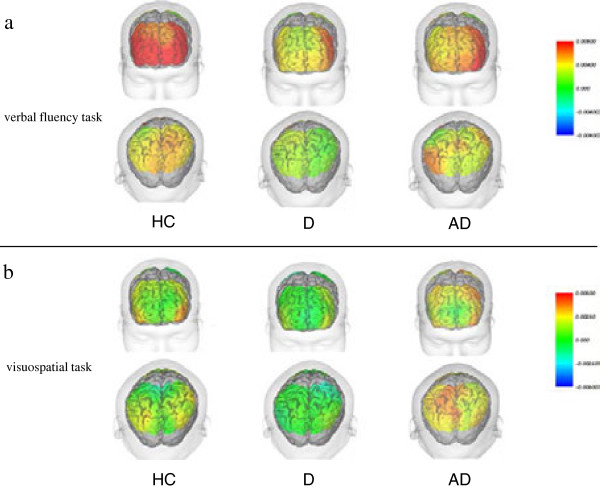
**General cortical activation in the three participant groups.** Superimposed images on 3-D MRI represent cortical activation in the verbal fluency task **(a)** and visuospatial task **(b)**. Upper figures show the activation of the frontal cortex, and lower figures show the activation of the parietal cortex. The color bar indicates [oxy-Hb] (mM·cm). Note that the scale of the color bar differs between figures **(a)** and **(b)**. Abbreviations: HC, healthy controls; D, depression; AD, Alzheimer’s disease; [oxy-Hb], oxygenated hemoglobin concentration.

Figure 1 shows the topography of cortical activation of each group.

#### Cortical activation in the VFT

**Figure 2:**
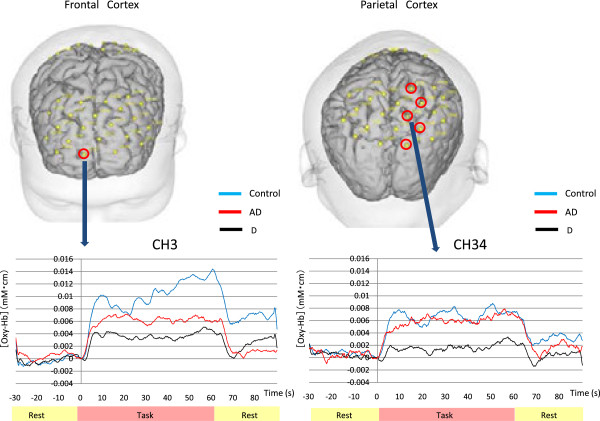
**Comparison of VFT results between the depressed group and healthy control.** Upper panels: The locations of the 44 channels on the head are indicated by yellow dots. The frontal cortex is on the left; the parietal cortex on the right. Significant differences are observed in 6 of the 44 channels indicated by red circles. Lower panels: Grand average waveforms of changes in [oxy-Hb] of CH 3 (left) and CH 34 (right) in the three groups, both of which show significant differences. Blue line, healthy controls; red line, AD group; black line, depressed group. In all three groups, during the rest period from -30 to 0 s, there are slight changes in [oxy-Hb], and during the task period from 0 to 60 s, [oxy-Hb] associated with brain activation is increased. At 60 s (when the task concluded), [oxy-Hb] is decreased. Abbreviations: D, depression; AD, Alzheimer’s disease.

The Kruskal-Wallis test revealed significant differences in CH 1 (p = 0.030), CH 2 (p = 0.050), CH 3 (p = 0.016), CH 12 (p = 0.029), CH 13 (p = 0.021), CH 30 (p = 0.014), CH 34 (p = 0.005), CH 35 (p = 0.001), CH 36 (p = 0.045), CH 39 (p = 0.022), and CH 43 (p = 0.031). The Mann-Whitney U-test showed significant differences only between the healthy controls and patients with depression. Cortical activation in the depressed group was decreased in comparison with healthy controls (Figure 1a). Significant differences were observed in CH3 of the frontal lobe (p = 0.043) and in CH 30 (p = 0.043), CH 34 (p = 0.041), CH 35 (p = 0.008), CH 39 (p = 0.043), and CH 43 (p = 0.043) of the right parietal lobe (p-values are FDR corrected) (Figure 2).

#### Cortical activation in the visuospatial task

**Figure 3:**
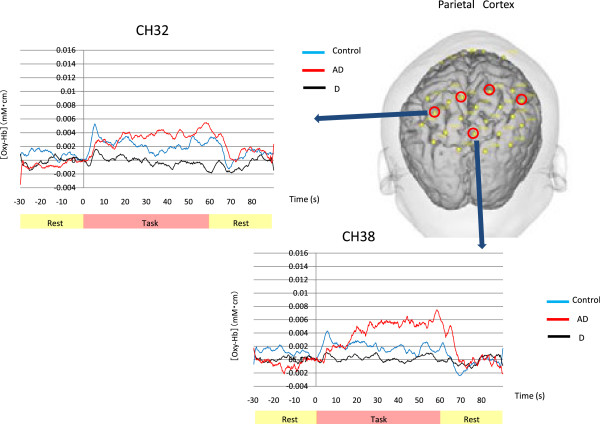
**Comparison of visuospatial task results between the depressed group and AD group.** Significant differences are seen in 5 of the 44 channels indicated by red circles. The graphs show the grand average waveforms of changes in [oxy-Hb] of CH 32 and CH 38 in the three groups, 2 of the 5 channels which showed significant differences.

The Kruskal-Wallis test revealed significant differences in CH 15 (p = 0.033), CH 17 (p = 0.046), CH 18 (p = 0.036), CH 24 (p = 0.006), CH 25 (p = 0.006), CH 26 (p = 0.017), CH 28 (p = 0.031), CH 31 (p = 0.002), CH 32 (p = 0.011), CH 34 (p = 0.039), CH 38 (p = 0.002), and CH 41 (p = 0.042). The Mann-Whitney U-test showed significant differences only between the depressed group and the AD group, where cortical activation was relatively decreased in the depressed group (Figure 1b). Significant differences were observed in CH 24 (p = 0.037), CH 25 (p = 0.020), CH 31 (p = 0.020), CH 32 (p = 0.035), and CH 38 (p =0.014) of the parietal lobe (p-values are FDR corrected) (Figure 3).

### Correlations between cortical activation and clinical data

Cortical activation did not correlate significantly with age, task performance, or score on the MMSE, FAB, CDR, or HAMD in any of the participant groups.

### Results of logistic regression analysis

The only channel with significance for predicting diagnosis during the visuospatial task was CH 38. Nagelkerke’s coefficient of determination showed a variance of 29.6% in the model. The total AUC was 0.769. The optimal cut-off point of cortical activation measured by CH 38, as determined using the Youden index, was 0.0015. The sensitivity and specificity for differentiating patients with depression from those with AD were 71.5% and 70.0%, respectively. Cortical activation during the VFT did not significantly predict diagnosis.

## Discussion

The objective of this study was to investigate the differences in brain activation between late life depression and AD. The results revealed that cortical activation in a visuospatial task was significantly lower in the parietal cortex of the depressed group than in that of the AD group. Similar but non-significant tendencies were seen in the VFT. Contrary to our hypothesis, parietal activation was lower in the depressed group than in the AD group. This was due to the fact that the activation was decreased in the depressed group in both the frontal and parietal cortices, while in the AD group, cortical activation was maintained. Figure 1 shows the general cortical activation patterns of the NIRS measurement area. Differences in cortical activation between the three groups were observed across the whole measurement area, suggesting an overall decrease in [oxy-Hb] change across the whole brain in patients with depression but not in those with AD. The cortical activation of the superior parietal lobe and precuneus was significantly higher in patients with AD than in those with depression. Superior parietal lobe is associated with spatial orientation and plays a role in maintaining the internal representation of the body's state [35]. Precuneus is involved in a variety of functions including visuo-spatial imagery, episodic memory retrieval, and self-processing operations [36]. The present results may reflect the difference in these functions between patients with AD and those with depression.

Previous NIRS studies have reported decreased frontal activation in patients with depression compared with healthy controls [6,9]. However, to our knowledge, no studies have examined the activation of the parietal cortex in patients with depression. The present study showed decreased activation in the parietal cortex, as well as in the frontal cortex. In the VFT, significant decreases were observed in the right parietal cortex in comparison with healthy controls.

On the other hand, cortical activation was maintained in the AD group. Parietal dysfunction in AD is observed from the initial phase of the disease [21-23]. As a result, parietal activation was expected to be more decreased in the AD group than in the depressed group, but we actually found that parietal activation was significantly decreased in the depressed group, despite the virtually equivalent task performance scores. Although NIRS measures changes in rCBF associated with neural activity, based on the theory of neurovascular coupling [37], we believe that changes in rCBF do not necessarily correspond to task performance and cognitive function in patients. Two fMRI studies demonstrated that the parietal region was more activated in normal individuals at high risk for AD than in low-risk individuals, although the level of task performance was identical in both groups [17,18]. Accordingly, these studies speculated that greater cognitive effort was required by the high-risk group to achieve the same level of performance as the low-risk group. A positron emission tomography activation study showed that brain activity may increase to compensate for the declined cognitive function in patients with AD [38]. In the present study, the AD group with decreased cognitive function may have required more cognitive effort to achieve the same task performance as the depressed group, possibly resulting in relatively greater parietal activation in the AD group.

According to the compensation hypothesis [39], older adults with impaired cognitive function perform equivalently to younger adults but display overactivation in the brain cortex at a lower level of task demand; at a higher level of task demand, the brain cortex becomes underactivated and the performance becomes impaired in older adults compared with younger adults. A similar mechanism may explain the difference in the cortical activation between the AD and depressed groups. The significant differences in cortical activation during the visuospatial task between these groups may be explained by overactivation of the brain in the cognitively-impaired AD group caused by the visuospatial task, which is a low-level task. The differences in cortical activation, however, cannot be completely explained by the compensation hypothesis, because neurovascular coupling may be disrupted in patients with AD [40,41].

Previous NIRS studies have reported that parietal activation was decreased in patients with AD compared with healthy controls [4,5]. In the present study, however, no significant differences were observed between the two groups. Such inconsistency may be due to the differences in the type and duration of the tasks performed, as well as disease severity. Furthermore, the significant difference in age between the patients with AD and healthy controls may have influenced the results, because neurovascular coupling is also known to be altered by normal aging [41]. Vermeij *et al.*[42] reported that the effects of aging on the time course of the hemodynamic response in the prefrontal cortex must be taken into account when interpreting the results of neuroimaging studies.

A few points could be improved in the present study. The first is that NIRS could have been used to measure blood flow in the scalp in addition to rCBF [43-45]. The extent to which scalp blood flow is included in our data is unknown; therefore, future studies should examine systemic parameters such as heart rate, blood pressure, end-tidal CO_2_, skin conductance, and scalp blood flow in order to separate the effects of systemic blood flow from NIRS signals due to neural activity [44,46]. Second, the effects of drugs cannot be ruled out. Previous studies have shown that acetylcholine-esterase inhibitors influence neurovascular coupling [47]. Other studies have reported that administration of antidepressants have influence on [oxy-Hb] changes [48-50]. However, a review of neuroimaging studies on bipolar disorder by Phillip *et al.*[51] reported neither significant nor ameliorative effects of psychotropic medications on abnormal structural and functional neuroimaging measures. Furthermore, several studies showed no significant correlations between cortical activation measured by NIRS and the dose of psychotropic medications [52-54]. Third, the participants in this study were not matched by age. We know that in the frontal cortex, the cortical activation patterns change with age [42,55]. However, in our data, no significant correlations were found between age and cortical activation, and the effects due to age differences are considered to be minor. Fourth, the effect of cerebrocortical atrophy in AD cannot be ruled out. When the layer of cerebrospinal fluid thickens due to cerebral atrophy, the sensitivity of NIRS measurements decreases [56]. Fifth, the depressed group comprised patients with partially or fully remitted symptoms of depression with low HAMD scores. It remains to be elucidated whether the present findings apply during severe depressive episodes. Lastly, depression rating scales validated for use with elderly patients, such as the Geriatric Depression Scale [57], are possible alternatives to the HAMD. Further studies are required to investigate these issues.

## Conclusions

NIRS revealed differences in brain activation between late life depression and AD. NIRS is a promising tool to assist in the differential diagnosis of these conditions.

## Abbreviations

AD: Alzheimer’s disease; ANOVA: Analysis of variance; AUC: Area under the receiver operating curve; CDR: Clinical dementia rating; CH: Channel; [deoxy-Hb]: Deoxygenated hemoglobin concentration; FAB: Frontal assessment battery; FDR: False discovery rate; fMRI: Functional magnetic resonance imaging; HAMD: Hamilton rating scale for depression; MMSE: Mini mental state examination; NIRS: Near-infrared spectroscopy; [oxy-Hb]: Oxygenated hemoglobin concentration; rCBF: Regional cerebral blood flow; VFT: Verbal fluency task.

## Competing interests

The authors declare that they have no competing interests.

## Authors’ contributions

HK, AR, and NA conceived and designed the experiments. HK and AR performed the experiments. HK, YK, and DS analyzed the data. NS, TO, TY, TH, SI, TT, HG, HN, TH, and SW collected the data and helped with clinical diagnosis. YK, DS, and NS helped to draft the manuscript. HK wrote the paper. All authors contributed to and have approved the final manuscript.
